# Prevalence of suspected hypertrophic cardiomyopathy or left ventricular hypertrophy based on race and gender in teenagers using screening echocardiography

**DOI:** 10.1186/1476-7120-8-54

**Published:** 2010-12-10

**Authors:** Mohammad Reza Movahed, Deborah Strootman, Sharon Bates, Sudhakar Sattur

**Affiliations:** 1 Sarver Heart Center, University of Arizona College of Medicine, Department of Medicine, Division of Cardiology, 1501 N Campbell Avenue, Tucson, Arizona, USA; 2 The Southern Arizona VA Health Care System, Department of Medicine, Division of Cardiology, Cardiology (1-111C), 3601 South Sixth Avenue, Tucson, AZ 85723, USA; 3 Anthony Bates Foundation, 428 E. Thunderbird, #633, Phoenix, AZ 85022, USA

## Abstract

**Background:**

The goal of this study was to evaluate the prevalence of suspected hypertrophic cardiomyopathy (HCM) in a population of teenagers undergoing screening echocardiography for the detection of HCM.

**Method:**

The Anthony Bates Foundation performs screening echocardiography for the prevention of sudden death. A total of 2,066 students were studied between the ages of 13 to 19 years. Suspected HCM was defined as any wall thickness ≥ 15 mm. LVH was defined as wall thickness ≥ 13 mm

**Results:**

Prevalence of suspected HCM was 0.7% (14/2066). After adjusting for hypertension (HTN), the total prevalence was 0.5% (8/1457). In a subgroup analysis, 551 teenagers with documented race and LV wall thickness were identified between the ages of 13 - 19 years. African American teenagers [6% (3/50)] had higher prevalence of suspected HCM [0.8% (4/501), OR 7.93, CI 1.72-36.49, p = 0.002]. After multivariate adjustment for age, gender, BMI and HTN (systolic BP >140 and diastolic BP of > 90), African American race remained independently associated with suspected HCM (OR 4.89, CI 1.24-39.62, p = 0.02).

**Conclusion:**

The prevalence of suspected HCM in young teenagers is approximately 0.2%. This prevalence appears to be higher in African Americans. However, due to small number of African Americans in our population, our result needs to be confirmed in larger trials.

## Introduction

Hypertrophic cardiomyopathy (HCM) is a common cause of sudden death in young people [1,2]. HCM is a relatively common genetic disorder with an estimated prevalence of 1 in 500 young adults [3]. Several periodic screening programs for early identification of HCM have been in practice across the country including Antony Bates Foundation and A Heart for Sport. HCM has been extensively studied for the last 40 years; however, there have been no prior studies in literature measuring prevalence of HCM in healthy teenagers prospectively in a large population.

We conducted this study to evaluate the prevalence of suspected HCM in a population of healthy teenagers who underwent voluntary health screening examination using echocardiography. We also evaluated for any race, gender and age range differences (13 -15 years vs. 16 -19 years) in the prevalence of HCM.

## Methods

The Anthony Bates Foundation has been conducting periodic health screening evaluations for high school students since 2001 for the detection of hypertrophic cardiomyopathy in early age. The evaluation includes history and physical examination, electrocardiogram and hand held echocardiogram. Board certified cardiologists reviewed these echocardiograms on site. Inform consent was obtained before the procedure. Participants were recruited using advertisement by the Antony Bates Foundation. Screening was limited to participants ≥ 12 years. Informed consent of teenagers younger than 18 was obtained from their parents.

The Anthony Bates Foundation targets young people in puberty. The program is voluntary. Participants are not required to be engaged in any athletic activities. The populations studied were volunteers screened for prevention of sudden death in young students. Although the screening was aimed for young subjects, no volunteer would be excluded for any reasons irrespective of age or comorbidities. This program was designated to report any suspected abnormal echocardiographic findings to the participant with recommendation to be followed by the participant's physician. Further follow up and work up was not included in the data base.

We used different models of hand held/mobile echo devices from Cypress (Siemen), Optigo (Philips), Vividi (GE), Sequoia (Simens) and SonoSite (GE). Different echo devices used for our study had different capabilities. However, for function and measurements used in our study, all echo devices used were appropriate and accurate. The hand held devices were all donated by the Regional Echocardiography Manufacturers Sales Force, local echocardiography companies, and participating hospitals during the screening.

Left ventricular septum and posterior wall were measured in the standard parasternal long axis view below the tip of the mitral valve. Furthermore, the entire left ventricle (LV) was routinely visualized to assess for localized hypertrophy. The correct position was confirmed by on site cardiologist. Wall thickness in end diastole was measured in the standard parasternal long axis view below the tip of the mitral valve and was confirmed by reading cardiologist at site. Other views were also reviewed to evaluate for occurrence of localized abnormal wall thickness. The obtained information is being continuously incorporated into a database.

Suspected HCM was defined as any wall thickness ≥15 mm based on the current guidelines[4]. Hypertension was defined as a systolic blood pressure > 140 mmHg or diastolic blood pressure > 90 mm. The diagnosis of hypertension was based on one blood pressure measurement during the screening. We used SPSS 15.0 to conduct statistical analysis. Height and weight were available which were used for BMI calculation. Obesity was defined in subjects with a BMI of over 30. We used univariate and multiple regression analysis adjusting for comorbidities. Furthermore, in order to evaluate for possible late expression of HCM genes in life, we compared the prevalence of HCM in two different age groups (13-16 and 16-19). Data analysis was performed in blinded fashion. A p value of less than 0.05 was deemed to be statistically significant. Multivariable analysis was performed in only a subset of patients due to the lack of data and this may lower the power of the model. Study period includes data from 2001-2009.

## Results

A total of 2,066 teenagers were identified between the ages of 13 - 19 years with documented left ventricular wall thickness. The total prevalence of suspected HCM, defined by a cut off value of 15 mm or more, was 0.7% (14/2066). After excluding teenagers with hypertension documented during screening, the prevalence of suspected HCM decreased to 0.5% (8/1457). There was no difference between the gender or age ranges using the cut off of ≥ 15 mm. We found 0.4% (3/688) of females vs. 1.1% (15/1377) of males had suspected HCM, p = 0.3 and 0.2% in teenagers between the age of 13-15 years vs. 0.3% between the age of 16-19 years, had suspected HCM, p = 0.7. Using a cut off value of 13 mm or more for the presence of left ventricular hypertrophy (LVH), [4] LVH occurred in 2.8% (57/2066) of the study population with a higher prevalence in male (3.6% in male vs. 1% in females, p = 0.001). Furthermore, African Americans had a higher mean posterior or septal wall thickness in comparison to other races (Table 1)

**Table 1:** Mean differences in between African American and other races.

	AA	Others	p
BMI	25.7 ± 4.8	23.4 ± 4.6	0.001
Age	17.0 ±1.6	16.3 ± 1.7	0.004
PWT D	0.96 ± 0.20	086 ± 0.28	0.015
SWTD	0.99 ± 0.21	0.85 ± 0.16	< 0.001
LVDED	4.8 ± 0.55	4.6 ± 0.58	0.026

AA = African Americans, BMI = Body Mass Index, PWTD = Posterior wall Thickness in Diastole, SWTD = Septal Wall Thickness in Diastole, LVEDD = Left Ventricular Dimension in End Diastole

### Sub group analysis showing race based prevalence

**Figure 1:**
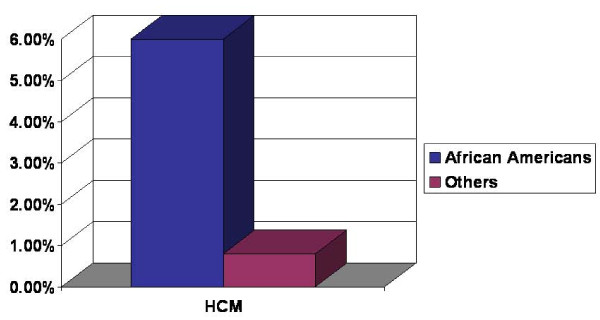
**Prevalence of Suspected Hypertrophic Cardiomyopathy (in percent) based on the African American race in the screened teenagers**.

A total of 551 teenagers with documented race demography and LV wall thickness were identified between the ages of 13 - 19 years. The estimated prevalence of HCM was 1.3% (7/551) in this population. African American teenagers [6% (3/50)] had higher prevalence compared to all other races [0.8% (4/501), OR 7.93, CI 1.72-36.49, p = 0.002] (Figure 1). The mean age of African American group was 17.0 ± 1.6 year vs. 16.3 ± 1.7 in all other races, p = 0.027. None of the African American subjects with suspected HCM had large left ventricular size over 5.7 cm. Overweight was more prevalent in African American race [(16.3% (8/49) vs. 9.8% (44/449) in all other races, p = 0.151)]. Only 12% (6/50) of the African American were female vs. 39% (196/503) of all other races (p < 0.001). After multivariate adjustment for age, gender, BMI and HTN (systolic BP >140 and diastolic BP of > 90), African American race remained independently associated with suspected HCM (OR 4.89, CI 1.24-39.62, p = 0.02). There was no significant difference in the prevalence of LVH or suspected HCM between other races.

## Discussion

Our study showed three important findings. First of all, the estimated prevalence of suspected HCM across the study population, after adjusting for hypertension, was 0.5%. Second, there was no significant gender difference and age range difference in the prevalence of HCM. African American race appears to have higher prevalence of HCM compared to all the other races independent of age, gender, BMI and HTN. However, we had small number of African Americans in our population, limiting our result that needs to be confirmed in larger studies.

The estimated prevalence of suspected HCM in our study is 1 in every 200 study subjects. Hada et al studied 12 000 adult Japanese workers initially with EKGs and subsequently with echocardiograms and reported prevalence of HCM was 0.2%[5]. A recent community-based study of patients referred for echocardiography because of a suspicion of cardiovascular disease demonstrated a 0.5% prevalence for HCM [5]. In a more recent echocardiogram based study of 4,111 young adults, Maron et al demonstrated a HCM prevalence of 0.2% (3). ECG based identification of HCM underestimates the prevalence of HCM compared to echo based studies. There is variability in the echo-based definition of HCM based on different HCM forms. In the consensus document HCM is defined if the septum thickness was >15 in the isolated form and >13 mm in the familial form [4].

In our study the prevalence increased to 2.8% from 0.5% when the echo definition of HCM was changed to ≥ 13 mm. Relaxing the cutoff for the wall thickness grossly overestimates the prevalence of HCM. Mildly increased LV wall thickness of 13 to 14 mm can also be seen in certain extreme expressions of the physiologically-based athletes heart [6-8] Our study has slightly higher prevalence than the general population probably secondary to above mentioned reasons.

Our study showed no significant gender differences in the prevalence of HCM. Similar findings were reported in prior studies [10,11]. Even though our study showed small numerical differences with increased males compared to females having HCM, the overall prevalence of condition is so low that it limits statistical power to identify any significant differences. There were also no significant differences in prevalence between the two age groups 13-15 vs. 16-19 years.

Over the years, HCM was considered an uncommon condition among the African American population. This was based on prior literature, which included relatively small number of African American patients[3,5,9-12]. Maron et al (2003), in a multi center analysis of clinically diagnosed HCM patients noted only 8% of the study population was black[12]. In our study, African Americans constitute only 9% of the population. Moreover, blacks had significantly higher prevalence of HCM compared to other races (6% vs. 0.8%). However, in the light of several premature cardiovascular deaths among African American athletes, Maron et al, in an autopsy analysis of young athletes surprisingly noted HCM was 7 times more commonly identified in African Americans for the first time at autopsy compared to when clinically identified in African American population[12]. Although African Americans constitute only 9% of our study population, they have a significant association with HCM using uni-variate and multi-variate analysis.

Even though African Americans constitute 16% of the population across United States, small numbers of African Americans participate in the screening events. Inadequate access to health care secondary to socio-economic reasons may contribute to decreased numbers of African Americans undergoing screening for diagnosis of HCM[13-15].

Our study focused on the age, gender and race based prevalence of suspected HCM in healthy teenagers between ages of 13-19 years. We conclude that prevalence of suspected HCM in the teenage population is similar to that reported in general population. African Americans have significantly higher prevalence of HCM compared to other races. A recent study reported that African American athletes exhibited greater LV wall thickness and cavity size compared to sedentary African Americans and Caucasians. In addition, the authors reported 3% of African Americanathletes in this study exhibited an LV wall thickness >= 15 mm, despite normal LV diastolic function and an enlarged LV cavity consistent with over diagnosis of HCM in this population[16]. These results could explain our finding of a higher prevalence of suspected HCM in our African American population. Therefore, it is important to recognize that the diagnosis of HCM in African Americans should be made with extreme caution based on LV wall thickness alone. It is possible that suspected HCM in our African American population does not represent true HCM. Data about LV volume in our subjects, which would have been useful in assessing the left ventricular dilation that could occur in the athletic heart, was not available for this study. However, we LV dimension was available as measured in standard parasternal long axis view. All African American subjects in our population with suspected HCM had normal left ventricular size suggesting that abnormal LV wall thickness was not related to athlete heart condition that should manifest as left ventricular dilatation in addition to left ventricular hypertrophy[17]. Furthermore, LV wall thickness greater or equal to 13 mm is very uncommon in highly trained athletes without cavity dilatation [8].

We suggest increased screening of African American teenagers for making early diagnosis of HCM by overcoming the possible challenges like lack of access to health care, which might help decrease morbidity and mortality associated with HCM in African American population. We also hope that our study will instigate further evaluation of the genetic make-up of HCM in African American population in order to better understand the disease and possible interventions to decrease associated morbidity and mortality.

Italian physicians have been the most experience with HCM screening programs. They have been successful in detecting early cardiac abnormalities including HCM in asymptomatic athletes [18-21]. For more than 20 years, 33,735 young athletes underwent screening leading to disqualification of athletes from training in 3.5% of participants[21]. None of the disqualified athletes died during a mean follow-up period of 8.2 years suggesting effective prevention. However, significant cardiac abnormalities in asymptomatic highly trained athletes have found to be extremely rare in England[22]. Future studies are needed to evaluate the cost effectiveness of routine screening for HCM in asymptomatic athletes.

## Limitations

Our study is a retrospective review of a data base. We used only echocardiographic measurement of wall thickness suspecting HCM. However HCM was not confirmed clinically or by further testing. Lack of significant differences in the prevalence of HCM between males and females could be due to small numbers of subjects and lack of sufficient statistical power. Our data base was acquired over many years and as time passed, new parameters were added to the questionnaire including adding more demographics such as race or gender which are not available in subjects with earlier screening date. Therefore, the number of subjects is lower in the multivariate analysis when adjusting for parameters that are not available in every participant limiting our multivariate analysis. The definition of hypertension was used using only single measurement of blood pressure. This may have increased the number of hypertensive subgroup. One of the major problems in the diagnosis of HCM is the differential diagnosis with LVH. We could not separate this two entities based on available data. This is the reason why we only used suspected HCM and not definite HCM in our manuscript. We have no outcome data as this study was performed at one time without follow up data availability. Our study is limited due to small number of African American participants but our results is highly significant and is consistent with previous reported studies that needs to be confirmed in larger trials.

## Competing interests

Anthony Bates Foundation founded screening and data collection

## Authors' contributions

MM carried out the statistical analysis and contributed to writing and formatting the manuscript including revisions. DS contributed to editing and data interpretation. SB performed screening and data collections. SS contributed to interpreting data and writing this manuscript. All authors read and approved the final manuscript version.
